# Analysis of proteome dynamics inside the silk gland lumen of *Bombyx mori*

**DOI:** 10.1038/srep21158

**Published:** 2016-04-22

**Authors:** Zhaoming Dong, Ping Zhao, Yan Zhang, Qianru Song, Xiaolu Zhang, Pengchao Guo, Dandan Wang, Qingyou Xia

**Affiliations:** 1 State Key Laboratory of Silkworm Genome Biology, Southwest University, 2, Tiansheng Road, Beibei, Chongqing 400716, China

## Abstract

The silk gland is the only organ where silk proteins are synthesized and secreted in the silkworm, *Bombyx mori*. Silk proteins are stored in the lumen of the silk gland for around eight days during the fifth instar. Determining their dynamic changes is helpful for clarifying the secretion mechanism of silk proteins. Here, we identified the proteome in the silk gland lumen using liquid chromatography–tandem mass spectrometry, and demonstrated its changes during two key stages. From day 5 of the fifth instar to day 1 of wandering, the abundances of fibroins, sericins, seroins, and proteins of unknown functions increased significantly in different compartments of the silk gland lumen. As a result, these accumulated proteins constituted the major cocoon components. In contrast, the abundances of enzymes and extracellular matrix proteins decreased in the silk gland lumen, suggesting that they were not the structural constituents of silk. Twenty-five enzymes may be involved in the regulation of hormone metabolism for proper silk gland function. In addition, the metabolism of other non-proteinous components such as chitin and pigment were also discussed in this study.

The silkworm, *Bombyx mori*, is one of the best-characterized silk-producing model organisms because its silk has great economic value. The composition of *B.* mori silk has been investigated intensively. Fibroin, the central fiber protein, is composed of heavy-chain proteins, light-chain proteins and p25^1^,^2^,^3^. Sericins are soluble glue proteins, coating and cementing the silk fibers, and three of them have been identified^4^,^5^,^6^. In a recent study, we revealed the complexity of the silk protein components by analysing seven silk proteomes^7^. In addition to the expected fibroins and sericins, we also identified various protease inhibitors, enzymes, proteins of unknown function and other proteins. Some of the identified silk proteins have definite roles in the silk, for example, protease inhibitors could protect the silk from degradation^8^,^9^. However, most proteins in the silk have unknown functions and need to be explored in depth.

Silk was spun efficiently at normal ambient temperatures and atmospheric pressure, which may be directly related to complex biochemical processes that occur in the silk gland. Silk glands expand rapidly as a result of DNA replication between day 1 and 5 in the fifth instar larvae, and complete the development on day 6 of the 5th instar^10^. Silkworm start to spinning on day 7 of the fifth instar (V-7), which is called wandering phase. Silk gland contain three compartments according its morphology and function, including the anterior silk gland, middle silk gland, and posterior silk gland. The fibroins and sericins are synthesized in the posterior and middle silk gland, respectively^11^,^12^,^13^,^14^, and then stored in the lumen of the silk gland as a concentrated aqueous silk solution^15^. When required for spinning, the proteins flow through the narrow anterior silk gland duct. In this process, the silk solution was converted into a solid filament by the combined action of strain and shear, together with the dehydration and induction of the ions^16^,^17^,^18^,^19^,^20^,^21^,^22^,^23^,^24^.

However, our knowledge on the process involved in the natural silk production is still incomplete. To extend our understanding, it is necessary to evaluate the molecular functions of various extracellular proteins in the silk gland lumen. Here, we identified the proteome in the silk gland lumen using liquid chromatography-tandem mass spectrometry (LC-MS/MS) and demonstrated its dynamic change in five compartments of the silk gland during two developmental stages. Therefore, this is a detailed study of the natural silk ‘production line’ in the perspectives of both biology and engineering.

## Results

### Extraction and identification of proteins in the silk gland lumen

**Figure 1:**
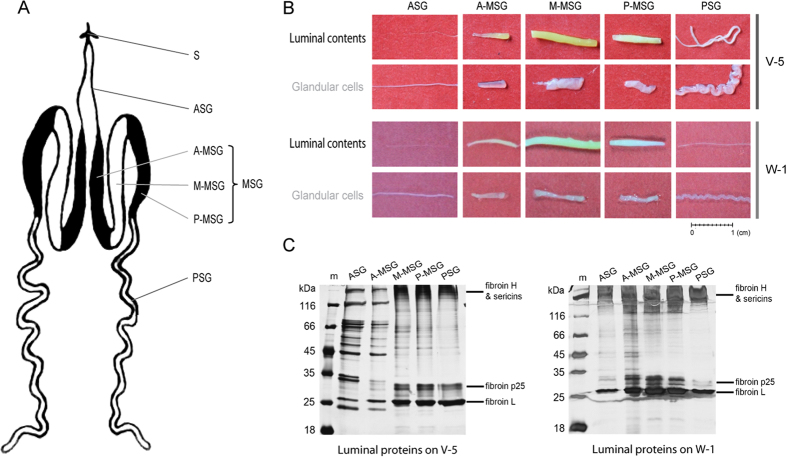
Extraction and electrophoresis of proteins in the silk gland lumen. (**A**) Schematic representation of the silk gland in the silkworm, *Bombyx mori*. S: spinneret; ASG: anterior silk gland; MSG: middle silk gland; PSG: posterior silk gland; A-MSG: anterior part of the middle silk gland; M-MSG: middle part of the middle silk gland; P-MSG: posterior part of the middle silk gland. (**B**) Photos of luminal contents and glandular cells of the silk gland. The silk gland was divided into five compartments according its morphology. The frozen luminal contents were extracted from the exterior silk gland cells at two stages: day 5 of the fifth instar (V-5) and day 1 of wandering (W-1). (**C**) SDS-PAGE of the luminal proteins in the silk gland.

The silk glands were dissected out from silkworms at two different developmental stages: day 5 of the fifth instar (V-5) and day 1 of wandering (W-1). Each silk gland was cut into five compartments according its morphology (Fig. 1A), including the anterior silk gland (ASG), the anterior part of middle silk gland (A-MSG), the middle part of the middle silk gland (M-MSG), the posterior part of the middle silk gland (P-MSG), and the posterior silk gland (PSG) (Fig. 1A). Each compartment was then divided into two parts, the glandular cells and the luminal contents (Fig. 1A,B). The luminal contents from different compartments showed a wide variety of diameters. In particular, the M-MSG had the maximum diameter of 2 mm, and the ASG had the minimum diameter of less than 0.1 mm (Fig. 1B). The luminal contents of the M-MSG was surrounded by a glandular cell “sheath” of a similar size, but the luminal contents of the ASG were much thinner than its glandular cell “sheath” (Fig. 1B), because the ASG contains a thick duct wall made of chitin which narrows the lumen^25^.

Results of SDS–PAGE revealed several similar, intensely-stained protein bands in the lumen of M-MSG, P-MSG, and PSG (Fig. 1C), which mainly included the 350 kDa fibroin heavy chain^26^,^27^, the 400 kDa or 150 kDa sericin1^6^,^28^, the 26 kDa fibroin light chain^29^, and the 27 kDa or 30 kDa fibroin p25 protein^1^,^2^,^3^. Fibroins and sericins were at low abundance in the lumen of ASG and A-MSG on day 5 of the fifth instar, but their level obviously increased on day 1 of wandering (Fig. 1C). A dozen protein bands seems similar between the ASG and A-MSG on day 5 of the fifth instar (Fig. 1C).

LC–MS/MS was used to determine the proteome in the lumen of five silk gland compartments during two developmental stages. With the combined analysis of triplicates samples, we identified 10,747 tryptic peptides, which assembled to 1271 proteins (Supplementary Dataset 1 and Dataset 2). The average number of peptides per protein was 8.5, leading to an average sequence coverage of 27.6% (Supplementary Dataset 2). Most proteins (94.2%) were identified by two or more unique peptides. On day 5 of the fifth instar, we identified 548, 446, 724, 797, and 645 proteins in the ASG, A-MSG, M-MSG, P-MSG, and PSG, respectively (Supplementary Figure S1). On day 1 of wandering, we identified 502, 839, 402, 387, and 716 proteins in the ASG, A-MSG, M-MSG, P-MSG, and PSG, respectively (Supplementary Figure S1). In contrast to day 5 of the fifth instar, substantial numbers of proteins increased in the A-MSG on day 1 of wandering, but decreased in the M-MSG and P-MSG on day 1 of wandering.

### Annotation of proteins in the silk gland lumen

**Figure 2:**
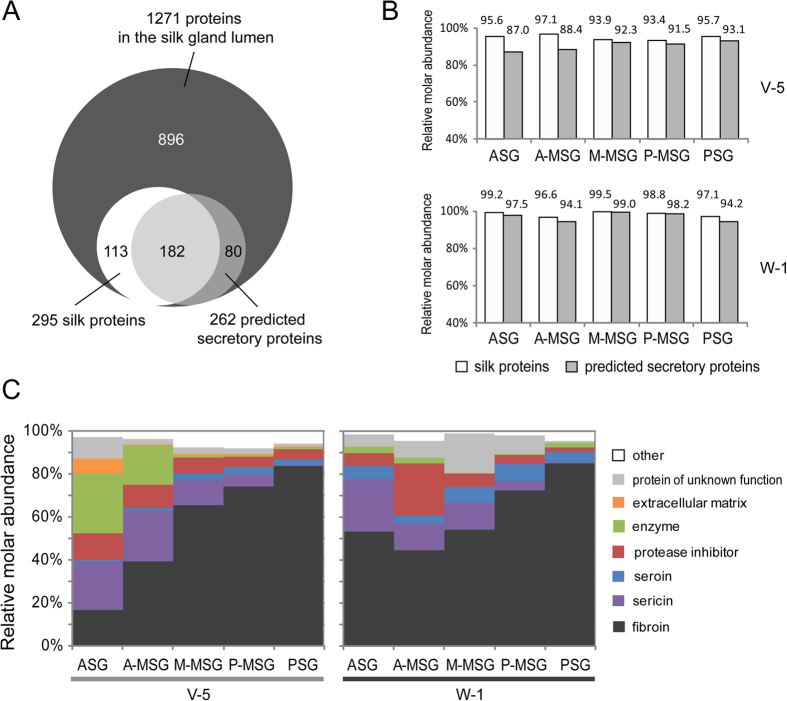
Identification, annotation and classification of proteins in the silk gland lumen. (**A**) LC-MS/MS identified 1271 proteins in the silk gland lumen, 295 proteins of them was identified as silk proteins (Dong *et al*. 2013; Zhang *et al*. 2015), and 262 proteins were predicted as secretory proteins by Phobius website (Supplementary Dataset 2). The secretory proteins contain signal peptides but no transmembrane regions. (**B**) The relative abundances of silk proteins and predicted secretory proteins in the lumen of silk gland. (**C**) The relative abundances of the proteins in the silk gland lumen according to their functional categories. The protein abundances were calculated with intensity-based absolute quantification (iBAQ).

The Blast2GO analysis tool (version 2.6.6)^30^ was used to subject the luminal proteins to GO annotation (Supplementary Dataset 2), and revealed that the identified proteins were involved in metabolic process, transcription and translation, ion transport, protein transport, stress response, signal transduction, extracellular matrix, regulation of proteolysis and cytoskeleton organization. The Phobius server was used to predict the transmembrane regions and signal peptides^31^, and identified 868 intracellular proteins, 262 extracellular proteins, and 141 transmembrane proteins (Supplementary Dataset 2). The identified intracellular proteins in the lumen of the silk gland might represent some leakage from cells of the silk gland. Although only 262 proteins were predicted to be extracellular proteins, they accounted for 87.0 ∼ 99.0% of the total protein abundance when being investigated with the intensity-based absolute quantification (iBAQ) intensity (Fig. 2A,B). Furthermore, we found that 295 proteins in the silk gland lumen were also detected in the scaffold silk and cocoon silk by previous studies^7^,^32^, making up 93.4 ∼ 99.5% of all protein molecules in the silk gland lumen (Fig. 2A,B).

Protein quantification results based on the functional classification indicated that proteins in the silk gland lumen mainly consist of fibroins, sericins, seroins, extracellular matrix proteins, protease inhibitors, enzymes, and proteins of unknown function, of which fibroins were the most abundant components (Fig. 2C and Supplementary Dataset 2). Proteins from each functional category increased or decreased in different compartments of the silkworm gland lumen form day 5 of the fifth instar to day 1 of wandering (Fig. 2C).

### Quantitative comparison of proteins in the silk gland lumen between two key stages

**Figure 3:**
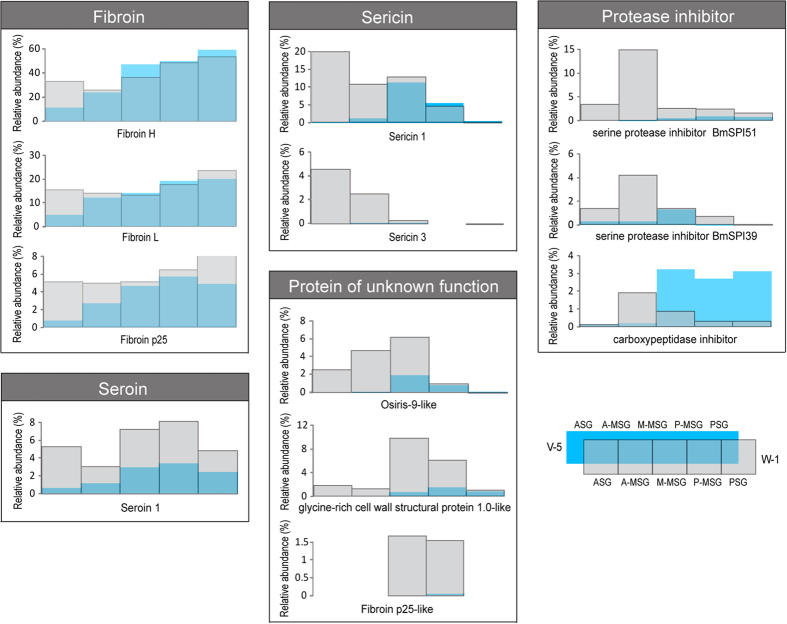
Constantly accumulated proteins in the silk gland lumen. Twelve proteins showed greatest increment from day 5 of the fifth instar to day 1 of wandering (Supplementary Table S1), which also constituted the twelve most abundant cocoon proteins (Zhang *et al*. 2015), including three fibroins, two sericins, one seroin, three protease inhibitors, and three proteins of unknown function. These proteins were compared according to their relative abundance (normalized iBAQ intensity) (Supplementary Dataset 2).

From day 5 of the fifth instar to day 1 of wandering, twelve proteins showed greatest increment based on intensities (Fig. 3 and Supplementary Table S1), including three fibroins, two sericins, three proteins of unknown functions, three protease inhibitors and one seroin. From day 5 of the fifth instar to day 1 of wandering, these twelve proteins increased in different compartments of the silk gland lumen (Fig. 3 and Supplementary Table S1): three fibroins (fibroin H, L and p25) jointly increased in the ASG, two sericins (sericin 1 and 3) increased in the ASG and A-MSG, three protease inhibitors (serine protease inhibitor BmSPI39 and BmSPI51, and carboxypeptidase inhibitor) mainly increased in the A-MSG, osifirs-9-like protein increased in ASG, A-MSG, and M-MSG, glycine cell wall structral protein 1.0-like protein and fibroin p25 like protein increased in the M-MSG and P-MSG, whereas seroin 1 increased in all the silk gland. As a result, these twelve proteins in the silk gland lumen constituted the twelve most abundant cocoon proteins, accounting 94.0 ∼ 96.8% of the total protein abundance^32^.

**Figure 4:**
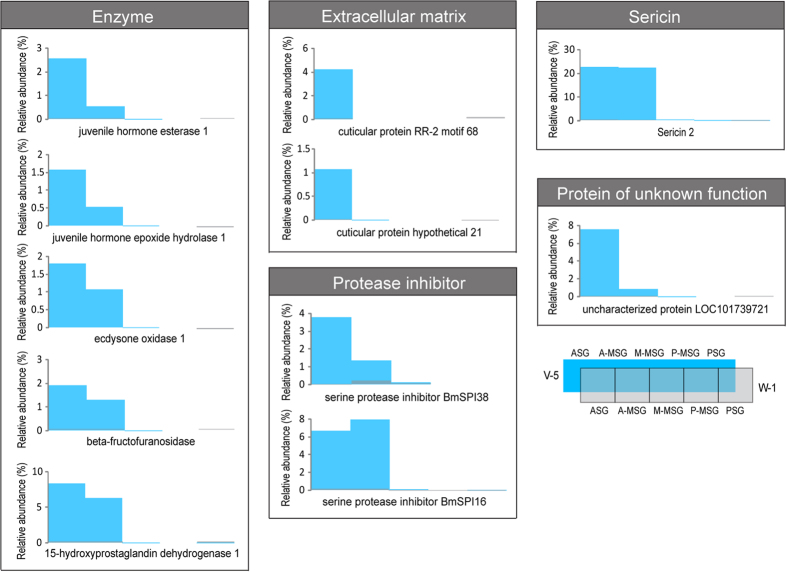
Drastically reduced proteins in the silk gland lumen. Eleven luminal proteins showed greatest reduction from day 5 of the fifth instar to day 1 of wandering (Supplementary Table S1), which were abundant in the ASG and A-MSG on day 5 of the fifth instar, but nearly disappeared on day 1 of wandering, including five enzymes, two extracellular matrix proteins, two protease inhibitors, one sericin and one protein of unknown function. These proteins were compared according to their relative abundance (normalized iBAQ intensity) (Supplementary Dataset 2).

From day 5 of the fifth instar to day 1 of wandering, eleven luminal proteins showed greatest reduction (Fig. 4 and Supplementary Table S1), which were abundant in the ASG and A-MSG lumen on day 5 of the fifth instar, but nearly disappeared on day 1 of wandering. All the eleven proteins significantly decreased (*P* < 0.05) in the ASG lumen, including five enzymes (juvenile hormone esterase 1, juvenile hormone epoxide hydrolase 1, ecdysone oxidase 1, beta-fructofuranosidase, 15-hydroxyprostaglandin dehydrogenase 1), two extracellular matrix proteins (cuticular protein RR-2 motif 68 and cuticular protein hypothetical 21), two protease inhibitors (BmSPI16 and BmSPI38), the sericin 2 and the uncharacterized protein (LOC101739721).

### Hormone metabolism enzymes were identified in the silk gland lumen

Hormones play important roles in the regulation of the development and function of silk gland^13^. Fortunately, we identified twenty-five enzymes in the silk gland lumen that may be involved in the hormone metabolism (Supplementary Table S2). Among them, eleven juvenile hormone esterase (JHE) and JHE-like proteins, three juvenile hormone epoxide hydrolases (JHEH) and JHEH-like proteins may be involved in the juvenile hormone metabolism, while eight ecdysone oxidase (EO) and EO-like proteins, one 3-dehydroecdysone 3 alpha-reductase (3DE-3α-R), and two 3-dehydroecdysone 3 beta-reductase (3DE-3β-R) like proteins may play roles in the molting hormone metabolism.

**Figure 5:**
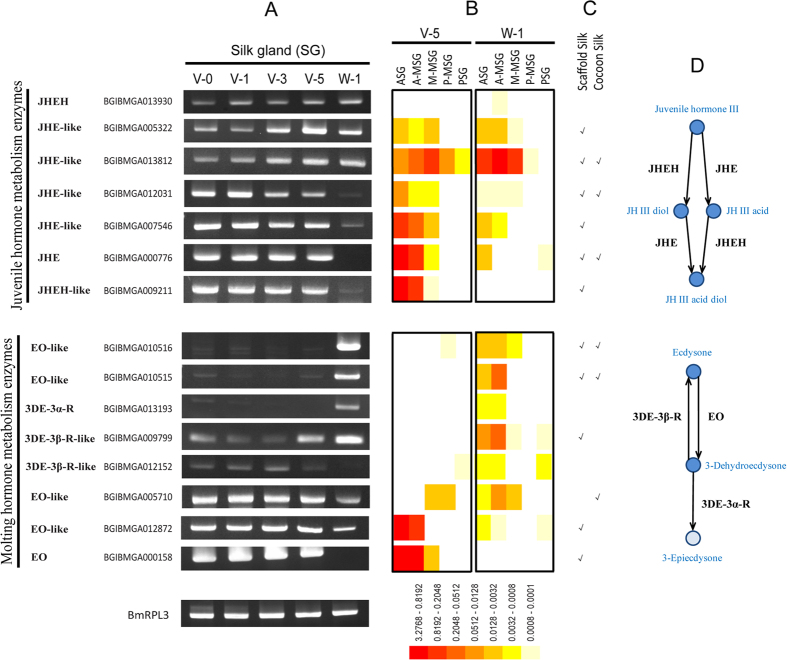
Hormone metabolism enzymes in the silk gland lumen. (**A**) Expression patterns of seven juvenile hormone metabolism enzymes and eight molting hormone metabolism enzymes. Semi-quantitative RT-PCR was performed using gene-specific oligonucleotides (Supplementary Table S3). The silkworm housekeeping gene ribosomal protein L3 (*BmRpl3*) was used as internal control (Liu *et al*. 2010). (**B**) The relative abundances of hormone metabolism enzymes in the silk gland lumen were compared according to the normalized iBAQ intensity (Supplementary Table S2). (**C**) Hormone metabolism enzymes detected in the scaffold silk and cocoon silk (Dong *et al*. 2013). (**D**) Hormone metabolism pathways in the silk gland lumen. This figure presents both the juvenile hormone metabolism pathway and molting hormone metabolism pathway according to the insect hormone biosynthesis pathways in the KEGG database and insect pathway database (iPathDB).

The results of semi-quantitative RT-PCR confirmed that fifteen hormone metabolism enzymes were expressed in the silk gland (Fig. 5A). It was noteworthy that day 5 of the fifth instar and day 1 of wandering were really two key stages, because that most of hormone metabolism enzymes had obvious expression change during the two stages (Fig. 5A). Their change in mRNA level was almost the same as the variation in protein level (Fig. 5B). Furthermore, protein quantification results suggested that most of hormone metabolism enzymes were secreted into the lumen of ASG and MSG (Fig. 5B), and twelve of which had been detected in the silk (Fig. 5C)^7^.

According to the insect hormone biosynthesis pathways in the KEGG database and insect pathway database^33^,^34^,^35^, we speculated that juvenile hormone (JH) may be metabolized to inactive JH acid, JH diol, and JH acid diol by JHE and JHEH, whereas ecdysone may be metabolized to 3-dehydroecdysone and 3-epiecdysone by EO and 3DE-3α-R in the silk gland lumen (Fig. 5D). It is a known fact that hormones control the development of silk gland and synthesis of silk^13^,^36^,^37^, whereas the hormone metabolism enzymes play important roles in the regulation of the hormonal titer^38^,^39^.

## Discussion

Silk proteins are synthesized and secreted by silk gland cells, and stored in the lumen of the silk gland for around eight days during the fifth instar. After that, silk proteins were spun out from the anterior silk gland to form silk fiber. Many silk-associated proteins may be involved in the silk production in the silk gland lumen. In this study, LC-MS/MS was used to elucidate the dynamic changes of silk-associated proteins in the silk gland lumen. This work revealed that some proteins constantly accumulated in the silk gland lumen during the fifth instar and constitute the major cocoon components, which include fibroins, secicins, antimicrobial proteins, and some proteins of unknown functions. In contrast, various enzymes and cuticular proteins tended to decrease in the silk gland lumen before spinning. The dynamic changes of silk-associated proteins in the silk gland lumen indicated that silk production is a quite complex process.

Fibroin heavy chain associated with the light chain fibroin and the P25 glycoprotein are produced by the PSG cells and constitute the core silk fiber. Besides fibroins, abundant seroin 1 were also identified in the PSG lumen, indicating that seroin 1 may have important functions in the PSG. Although seroin1 was reported to play antimicrobial roles^40^, it may have other functions, possibly similar to that of p25 protein^41^, playing a role to assemble fibroin heavy chains and light chains into an elementary silk unit^1^. When the fibroins flow into the MSG lumen, several layers of sericins are subsequently added to the fibroin core. Sericin P (150 kDa), sericin M (400 kDa) and sericin A (250 kDa) was identified in the P-, M- and A-MSG sections, respectively^28^. Thus, they correspond to the internal layer, middle layer and external layer of sericin, respectively^6^. Sericin M and sericin P were identified as products of the *sericin 1* gene and sericin A was found as the product of *sericin 3* gene^6^,^42^. Sericin 2, unlike the sericin 1 and 3, decreases on day 1 of wandering, which may be pushed into the spinneret by pressure from the accumulated proteins in the lumen. This speculation is consistent with its location and function: sericin 2 was the major coating proteins of non-cocoon silk, which was detected in the scaffold silk, the silk spun before cocoon construction^5^,^7^,^43^.

Three proteins with unknown functions were identified as the major cocoon proteins, which have similar sequence characteristic with fibroins but distinct spatial distribution. Glycine-rich cell wall structural protein 1.0-like is rich in glycine (36.5%) and alanine (12.5%) residues, liking the fibroin heavy chain, but has small protein size as 18.5 kDa. Osiris-9 like protein is rich in leucine (15.9%) and alanine (10.5%). Fibroin p25-like protein showed 53% identities with the fibroin p25. From day 5 of the fifth instar to day 1 of wandering, glycine-rich cell wall structural protein 1.0-like increased in the M-MSG lumen, fibroin p25-like protein increased in the M-MSG and P-MSG lumen, whereas osiris-9 like protein increased in the ASG, A-MSG and M-MSG lumen (Fig. 3). All the three proteins were identified as the major cocoon components^32^, but have uncharacterized functions.

Two protease inhibitors BmSPI51 and BmSPI39 increased before spinning, and mainly distribute in the A-MSG, corresponding to the external sericin layer. Previous studies found that BmSPI51 had high inhibitory activity against animal trypsin^8^, and its homolog in *Galleria mellonella* could inhibit both the trypsin and fungal proteases (subtilisin and proteinase K)^9^. BmSPI39 could significantly inhibit fungal proteases and spore germination of *Beauveria bassiana*^44^. Moreover, a recent study proved the protease inhibitors in the cocoon could inhibit trypsin and proteinase K but not chymotrypsin and elastase^32^, implied that they may be important in preventing the cocoon destruction by trypsin and fungal proteases. More protease inhibitors in the external sericin layer allows the cocoon to provide better protection from the predatory invasion.

Inside the ASG lumen, the chitin and chitin-binding cuticular proteins form a extracellular matrix layer to protect the gland cells from being damaged by high mechanical shear of spinning^7^,^25^,^45^, whereas shear force is important to induce self-assembly of silk proteins into fibrils^46^. The decrease of cuticular proteins on day 1 of wandering may reflect that incompact cuticular proteins have been assembled into a tough protective layer by cross-linking and binding to chitin^25^. Oxidases are responsible for the cross-linking of cuticular proteins^47^,^48^, while protease and chitinase may be involved in the degradation of extracellular matrix layer^7^. One recent study found that a serpin-type protease inhibitor BmSPI16 can regulate the activities of cysteine proteases in the silk gland^49^. Therefore, we speculated that some protease inhibitors, such as BmSPI16 and BmSPI38, may play roles to protect stored silk proteins inside the lumen from unexpected degradation. From day 5 of the fifth instar to day 1 of wandering, some protease inhibitors such as BmSPI16 and BmSPI38 decreased, little of which could be detected in the cocoon, whereas some protease inhibitors such as BmSPI51 and BmSPI39 increased and became the major cocoon components. It may be due to that they have divided roles to protect the silk proteins in the silk gland or in the cocoon.

Besides chitin, other non-proteinous components such as pigments are also actively secreted in the silk gland, and accumulate in the sericin layers of cocoon. The cocoon pigments vary depending on the *B. mori* strain. Some silkworm strains produce yellow-green cocoon shells were found containing flavonoid pigments^50^, including the DaZao strain used in this study^51^. Flavonoid pigments could be observed in the middle silk gland of Dazao strain as in Fig. 1B. The flavonoids in the mulberry leaves were absorbed by the silkworm midgut, and then glucosylated in the midgut and silk gland^52^. In this study, four UDP-glucosyltransferase were identified in the silk gland, which could transfer glucose to the hydroxyl groups of flavonoids^52^. The mechanisms for transport of flavonoids are not well understood in any animal system, but the glucosylation at the 5-O position was speculated as the key step to allow or facilitate the efficient uptake and transport of flavonoids from midgut to silk gland^53^. The glucosylated flavonoids may be used to increase the anti-oxidative state of the tissues and increase the UV-shielding activity of cocoons^52^,^53^.

Twenty-five enzymes identified in this study may be involved in the hormone metabolism. Both the JHE and JHEH are responsible for the degradation of JH (Fig. 5D). JHE and JHE-like genes continuously expressed in the silk gland from day 0 of the fifth instar to day 5 of the fifth instar (Fig. 5A), which were considered to be controlled by hemolymph JH titer^38^. The expression of JHEH and JHEH-like genes are probably regulated in similar manners, because their expression profiles are similar to that of JHE and JHE-like (Fig. 5A). On day 1 of wandering (day 7–8 of the fifth instar), some JH metabolic enzymes were down-regulated at both the mRNA and protein level (Fig. 5A), which may be due to the rise of ecdysteroids^38^. The secretory JHE in the hemolymph were reported in previous studies^54^,^55^,^56^, whereas the secretory JHE and JHEH in the silk gland lumen were found for the first time. We can not rule out the possibility that the detection of these enzymes in the silk gland lumen might due to their leakage from silk gland cells^7^. However, six of the JH metabolic enzymes were predicted to have signal peptides, indicated that they may have roles in the extracellular matrix to degrade JH around cells^38^.

EO and 3DE-3α-R play roles to degrade the molting hormone (ecdysteroids), whereas 3DE-3β-R is involved the biosynthesis of ecdysteroids (Fig. 5D). The ecdysteroids maintain low level during the feeding stage, which is necessary for proper silk gland function^37^,^57^. With the sharp rise of ecdysteroids on day 1 of wandering, ecdysteroids metabolic enzymes showed corresponding fluctuation, decrease or increase, in the silk gland (Fig. 5A). Two EOs were identified in the silkworm^39^,^58^, both of which belong to the family of glucose–methanol–choline oxidoreductase. Sun *et al*. considered that BmEO/BmGMC2 (BGIBMGA000158) may have not EO activity, because it only has 1 of 5 conserved ecdysone-binding residues^39^. Therefore, the activities of these putative ecdysteroids metabolic enzymes are still to be validated in future.

## Methods

### Sample collection and electrophoresis

The silkworm strain DaZao was reared on mulberry leaves at 25 °C. The silk glands were dissected out at 4 °C in 0.75% (w/v) NaCl on day 5 of the fifth instar and day 1 of wandering, before spinning. They were frozen in liquid nitrogen for 2 min and then immersed in 60% pre-cool ethanol for 2 min. The silk gland was divided into five compartments: ASG, A-MSG, M-MSG, P-MSG, and PSG (Fig. 1A). The solid luminal contents were then dragged out of the exterior silk gland cells. The luminal contents from twenty individuals were collected as one sample, and were dissolved in 9 M LiSCN with vortexing for 2 h. The solubilised proteins were recovered by centrifugation (12,000 g, 10 min, 4 °C). The protein concentrations in the supernatants were determined with the Bradford method^59^. Equal amounts (2 μg) of luminal proteins were separated on 12.5% (w/v) polyacrylamide gel and visualized with silver staining.

### Protein digestion and LC-MS/MS

The luminal proteins (60 μg) were digested according to previously reported methods^7^,^32^,^60^,^61^. The resulting tryptic peptides were recovered by centrifugation in the ultrafiltration tube, lyophilized, and resuspended in 35 μL of 0.1% formic acid. The tryptic peptides (6.5 μL) were separated on the Thermo Fisher Scientific EASY-nLC 1000 system using a Thermo Fisher Scientific EASY-Spray column, with a 120 min gradient consisting of 2 min at 3%–8% buffer B (100% acetonitrile, 0.1% formic acid), 80 min at 8%–20% buffer B, 10 min at 20%–30% buffer B, 5 min at 30%–70% buffer B, 3 min at 70%–90% buffer B, and 20 min at 90% buffer B. The separated peptides were analysed with a Thermo Scientific Q Exactive Hybrid Quadrupole-Orbitrap mass spectrometer operating in data-dependent mode. The instrument parameters were as follow: the resolution was 70,000 for full MS scan and 17,500 for MS^2^ scan; the automatic gain control target was 3E6 for full scan and 1E6 for MS^2^; the maximum ion injection time was 20 ms for full MS scan and 60 ms for MS^2^ scan. Three biological replicates were used for the LC-MS/MS analyses.

### Protein identification, quantification and annotation

The resulting raw MS data were analysed with the MaxQuant software (version 1.3.0.1)^62^. The MaxQuant searches were executed against an integrated silkworm proteome database containing 35,379 protein sequences from NCBI and silkDB. Peptide searches were performed with the Andromeda search algorithms^63^. The search parameters were set as reported previously^7^,^32^,^60^,^61^. A minimum of one unique peptides was required for the identified protein. All common contaminants and reverse hits were removed. The identified peptides and proteins are listed in Supplementary Dataset 1 and Dataset 2, respectively.

The iBAQ algorithm in MaxQuant was used to compare the protein abundances^64^. We assumed that the total intensity of each sample was same (its intensity was set as 100%), and then normalized the relative intensity of each protein. The estimates of protein intensity are presented in Supplementary Dataset 2. Heat map of protein abundance was generated using the HemI (Heatmap Illustrator, version 1.0.3.3)^65^. Two-tailed t-test was used to determine the differential expression between day 5 of the fifth instar and day 1 of wandering.

To annotate the molecular functions of proteins, we used the Blast2GO software (version 2.6.6)^30^, an all-in-one program for performing Blast searches, Gene ontology (GO) annotation, enzyme code (EC) annotation, signal peptides prediction, transmembrane domain prediction and KEGG pathway construction. The default settings of Blast2GO were used in every step.

### Temporal expression analysis of hormone metabolism enzymes in the silk gland

The silk glands were collected at five different developmental stages: day 0 of the fifth instar, day 1 of the fifth instar, day 3 of the fifth instar, day 5 of the fifth instar, day 1 of wandering. Total RNA was isolated using TRIzol reagent (Invitrogen, USA). Contaminating genomic DNA was digested using RNase-free DNase I (Promega) for 30 min at 37 °C. Total RNA (10 μg) was reverse-transcribed into cDNA using M-MLV reverse transcriptase (Invitrogen, USA) at 42 °C. All cDNA samples were normalized using *B. mori* housekeeping gene ribosomal protein L3 (*BmRPL3*) as an internal control (forward primer: 5′-TCG TCA TCG TGG TAA GGT CAA-3′; reverse primer: 5′-TTT GTA TCC TTT GCC CTT GGT-3′)^66^. The primers for semi-quantitative RT-PCR detection are listed in Supplementary Table S3. PCR amplification was performed in a total reaction volume of 25 μL using the following program: initial incubation at 94 °C for 4 min, followed by 28 cycles of 40 s at 94 °C, 40 s of annealing (52–58 °C), 30–50 s of extension (72 °C), and a final extension at 72 °C for 10 min. Aliquots of 5 μL of the PCR products were separated on 1.3% agarose gels and stained with EB.

## Additional Information

**How to cite this article**: Dong, Z. *et al*. Analysis of proteome dynamics inside the silk gland lumen of *Bombyx mori. Sci. Rep.* **6**, 21158; doi: 10.1038/srep21158 (2016).

## Supplementary Material

Supplementary Information

Supplementary Dataset
