# Supplementary material for: Analysis of proteome dynamics inside the silk gland lumen of Bombyx mori
Source: Sci Rep. 2016 Apr 22;6:21158. doi: 10.1038/srep21158 (PMC4840313; doi:10.1038/srep21158)
Supplement: Supplementary Information [file srep21158-s1.pdf]

Analysis of proteome dynamics inside the silk gland lumen of *Bombyx mori*

Zhaoming Dong, Ping Zhao, Yan Zhang, Qianru Song, Xiaolu Zhang, Pengchao Guo, Dandan Wang, Qingyou Xia

State Key Laboratory of Silkworm Genome Biology, Southwest University, 2, Tiansheng Road, Beibei, Chongqing 400716, China

Corresponding author: Qingyou Xia

E-mail: xiaqy@swu.edu.cn

Supplementary Figure S1

Dynamic changes of proteins in the silk gland lumen. This heat map was generated using the HemI (Heatmap Illustrator, version 1.0.3.3). The protein abundances were showed by log2-transformed intensity-based absolute quantification (iBAQ) intensities. Protein abundances were represented by red (higher) and green (lower) boxes. Missing data were represented by white. The columns represented five silk gland compartments of two stages.

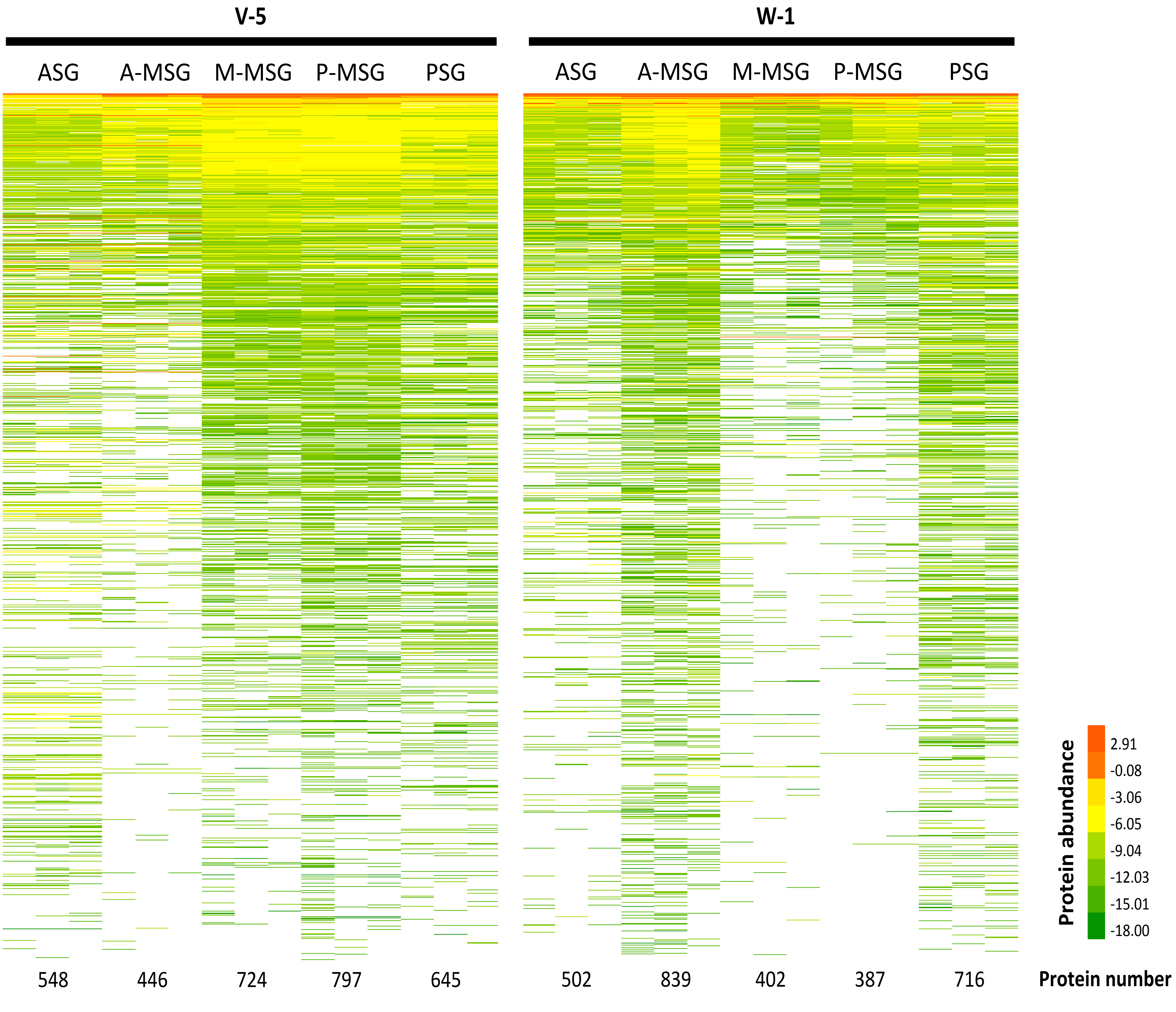

Supplementary Table S1. Proteins in the silk gland lumen that showed greatest change.

Classsification	Protein IDs	Seq. Description	Signal peptides	Unique peptides	Mol. weight [kDa]	Average iBAQ V-5 ASG	Average iBAQ V-5 A-MSG	Average iBAQ V-5 M-MSG	Average iBAQ V-5 P-MSG
fibroin	>gi|164448672|ref|NP_0011(	fibroin heavy chain	Yes	22	391.59	11.2023	23.5259	46.5913	49.3623
fibroin	>BGIBMGA009393-PA pep:	fibroin light chain	Yes	2	27.669	4.89483	12.2029	14.2716	19.1357
fibroin	>BGIBMGA001347-PA pep:	fibroin p25	Yes	4	25.149	0.75448	2.69821	4.67651	5.72846
sericin	>gi|66774164|sp|P07856.2|SI	sericin 1	Yes	33	119.48	0.15925	1.35464	11.4649	5.61292
sericin	>gi|282848475|gb|ADA8414'	sericin 2	Yes	22	198.65	22.5824	22.4088	0.32582	0.00131
sericin	>gi|157889130|dbj|BAF8102	sericin 3	Yes	61	123.3	0	0.02063	0.01202	0
seroin	>BGIBMGA002689-PA pep:	seroin 1	Yes	11	7.6522	0.624	1.14196	2.94803	3.37803
protein of unknown function	>BGIBMGA001358-PA pep:	glycine-rich cell wall structural protein 1.0-like	Yes	5	18.534	0	0	0.72146	1.44464
protein of unknown function	>gi|512891160|ref|XP_00492	fibroin p25-like	Yes	17	27.201	0	0	0	0.03285
protein of unknown function	>BGIBMGA000013-PA pep:	osiris 9-like	Yes	23	25.909	0	0.00153	1.87315	0.72017
protein of unknown function	>gi|512894896|ref|XP_00492	uncharacterized protein LOC101739721	Yes	16	32.624	7.60583	0.83066	0.00344	0
protease inhibitor	>gi|112983122|ref|NP_00103	serine protease inhibitor kunitz type BmSPI51	Yes	4	8.4446	0	0.02622	0.48875	0.84577
protease inhibitor	>gi|164448666|ref|NP_0011(	carboxypeptidase inhibitor	Yes	9	12.561	0.112	0.20045	3.23185	2.72701
protease inhibitor	>gi|512898429|ref|XP_00492	serine protease inhibitor TIL-type BmSPI39	Yes	7	22.654	0.29941	0.27594	1.33397	0.00612
protease inhibitor	>BGIBMGA009094-PA pep:	serine protease inhibitor TIL-type BmSPI38	Yes	4	8.765	3.82442	1.36325	0.12153	0
protease inhibitor	>BGIBMGA003292-PA pep:	serine protease inhibitor serpin-type BmSPI16	Yes	27	44.367	6.73155	7.9832	0.0608	0
extracellular matrix	>BGIBMGA010231-PA pep:	cuticular protein RR-2 motif 68	Yes	14	31.754	4.2265	0	0	0
extracellular matrix	>BGIBMGA011721-PA pep:	cuticular protein hypothetical 21	Yes	1	14.647	1.06957	0.00067	0	0
Enzyme	>BGIBMGA009211-PA pep:	juvenile hormone epoxide hydrolase 1	Yes	4	57.55	1.58481	0.53185	0.00065	0
Enzyme	>BGIBMGA000158-PA pep:	glucose dehydrogenase/ecdysone oxidase 1	Yes	36	61.805	1.81478	1.08039	0.0087	0
Enzyme	>gi|512903792|ref|XP_00492	carboxylesterase/juvenile hormone esterase 1	Yes	26	64.261	2.58311	0.54398	0.00203	0
Enzyme	>BGIBMGA005696-PA pep:	beta-fructofuranosidase	Yes	29	55.941	1.92511	1.30775	0.00138	0
Enzyme	>BGIBMGA005827-PA pep:	15-hydroxyprostaglandin dehydrogenase 1	Yes	5	31.689	8.42937	6.31263	0.02508	0

Average iBAQ V-5 PSG	Average iBAQ W-1 ASG	Average iBAQ W-1 A-MSG	Average iBAQ W-1 M-MSG	Average iBAQ W-1 P-MSG	Average iBAQ W-1 PSG	Increment of ASG (%)	Increment of A-MSG (%)	Increment of M-MSG (%)	Increment of P-MSG (%)	Increment of PSG (%)	*P* value of ASG	*P* value of A-MSG	*P* value of M-MSG	*P* value of P-MSG	*P* value of PSG
58.7832	33.454	26.3769	36.468	49.1513	54.0121	22.2517	2.85097	-10.123	-0.21101	-4.77117	0.00003164	0.26472457	0.00705742	0.91786041	0.04423657
20.0815	15.2222	13.723	12.8493	17.2044	23.243	10.3273	1.52014	-1.4224	-1.93126	3.16155	0.00001879	0.25261101	0.02415370	0.13340324	0.10568031
4.91792	4.96875	4.86664	4.99928	6.30386	8.13544	4.21428	2.16843	0.32277	0.5754	3.21753	0.00000581	0.16087441	0.58123745	0.35929723	0.00088454
0.44859	19.4926	10.5391	12.463	4.38027	0.00538	19.3333	9.18442	0.9981	-1.23265	-0.44321	0.00002002	0.00264770	0.29538398	0.09485384	0.00000002
0.00873	0.40636	0.07381	0	0	0.16825	-22.176	-22.335	-0.3258	-0.00131	0.15951	0.00000027	0.00001094	0.00014480	0.03436757	0.00844351
0	4.51428	2.46146	0.27159	0	0.00013	4.51428	2.44083	0.25956	0	0.00013	0.00185330	0.01499474	0.00067326	—	0.12167875
2.44471	5.20269	3.02172	7.11501	7.97551	4.78194	4.57869	1.87976	4.16698	4.59748	2.33723	0.00005390	0.00128920	0.00543385	0.00423656	0.00040852
0.75841	1.81354	1.17735	9.6001	5.9768	1.0318	1.81354	1.17735	8.87864	4.53216	0.27339	0.06179071	0.08632440	0.00097179	0.18377742	0.62954080
0	0	0	1.64942	1.52271	0	0	0	1.64942	1.48986	0	—	—	0.00184889	0.00030836	—
0.00197	2.50557	4.59	6.09109	0.97692	7.9E-05	2.50557	4.58848	4.21795	0.25675	-0.00189	0.00000089	0.00043607	0.00002915	0.21277500	0.12899186
0	0.00117	5.7E-05	0	0	0.00113	-7.6047	-0.8306	-0.0034	0	0.00113	0.00000959	0.00054399	0.00789436	—	0.04374748
0.69372	3.23595	14.5549	2.51358	2.35571	1.57569	3.23595	14.5287	2.02484	1.50994	0.88196	0.00130531	0.02119380	0.02040723	0.00186983	0.01927212
3.14308	0.09551	1.86435	0.8486	0.27663	0.30257	-0.0165	1.6639	-2.3833	-2.45038	-2.84051	0.29163047	0.00005288	0.00045303	0.00286762	0.00000575
0	1.32322	4.05566	1.30174	0.63902	0.00129	1.0238	3.77972	-0.0322	0.63291	0.00129	0.01606950	0.00063481	0.94395639	0.00458520	0.25041215
0	0.0042	0.17221	0.05602	0	0	-3.8202	-1.191	-0.0655	0	0	0.00000574	0.00813691	0.02307520	—	—
0.00013	0.11842	0.13486	0.00048	0.00193	0.01733	-6.6131	-7.8483	-0.0603	0.00193	0.0172	0.00000001	0.00001830	0.00028650	0.00001581	0.00344366
0	0.00296	0	0	0	0.09309	-4.2235	0	0	0	0.09309	0.00000370	—	—	—	0.00004432
0	0.01961	0	0	0	0.0008	-1.05	-0.0007	0	0	0.0008	0.00220550	0.37390097	—	—	0.15472160
0	0	0	0	0	0.00012	-1.5848	-0.5319	-0.0006	0	0.00012	0.00000034	0.00041892	0.00186571	—	0.37390097
0	0	0	0	0	0.0001	-1.8148	-1.0804	-0.0087	0	0.0001	0.00000054	0.00011032	0.00030352	—	0.14106875
0	0.00349	2.3E-05	0	0	0.00042	-2.5796	-0.544	-0.002	0	0.00042	0.00000326	0.00384795	0.00019838	—	0.00885740
0	0.00417	0.00054	0	0	0.00105	-1.9209	-1.3072	-0.0014	0	0.00105	0.00000405	0.00001261	0.00241896	—	0.00336939
0.0001	0.00508	0.00017	0	0	0.01748	-8.4243	-6.3125	-0.0251	0	0.01738	0.00000031	0.00119267	0.00041993	—	0.01305040

Supplementary Table S2. Hormone metabolism enzymes in the silk gland lumen.

Classsification	Protein IDs	Seq. Description	Transmembrane region	Signal peptides	Peptides	Razor + unique peptides	Unique peptides
juvenile hormone metabolism enzymes	>BGIBMGA013812-PA pep	juvenile hormone esterase-like protein 2	_	Yes	45	45	43
juvenile hormone metabolism enzymes	>BGIBMGA012031-PA pep	juvenile hormone esterase-like protein 7	_	Yes	20	20	19
juvenile hormone metabolism enzymes	>BGIBMGA007546-PA pep	juvenile hormone esterase-like protein 3	_	Yes	25	25	24
juvenile hormone metabolism enzymes	>gi|512903792|ref|XP_00492	juvenile hormone esterase	_	Yes	33	33	26
juvenile hormone metabolism enzymes	>BGIBMGA000775-PA pep	juvenile hormone esterase-like protein 6	_	_	18	12	3
juvenile hormone metabolism enzymes	>gi|512931403|ref|XP_00493	juvenile hormone esterase-like protein 5	_	_	21	21	21
juvenile hormone metabolism enzymes	>gi|512903970|ref|XP_00492	juvenile hormone esterase-like protein 8	_	_	12	3	3
juvenile hormone metabolism enzymes	>BGIBMGA010976-PA pep	juvenile hormone esterase-like protein 4	_	_	33	32	2
juvenile hormone metabolism enzymes	>gi|512934081|ref|XP_00493	juvenile hormone esterase-like protein 9	_	_	12	11	11
juvenile hormone metabolism enzymes	>gi|114052306|ref|NP_00104	juvenile hormone esterase-like protein 10	_	_	10	10	9
juvenile hormone metabolism enzymes	>BGIBMGA007670-PA pep	juvenile hormone esterase-like protein 11	Yes	_	4	4	4
juvenile hormone metabolism enzymes	>BGIBMGA013930-PA pep	juvenile hormone epoxide hydrolase	_	Yes	4	4	4
juvenile hormone metabolism enzymes	>BGIBMGA009211-PA pep	juvenile hormone epoxide hydrolase-like protein 2	_	Yes	18	18	4
juvenile hormone metabolism enzymes	>BGIBMGA011438-PA pep	juvenile hormone epoxide hydrolase-like protein 3	_	_	9	9	9
ecdysone metabolism enzymes	>BGIBMGA000158-PA pep	ecdysone oxidase	_	Yes	36	36	36
ecdysone metabolism enzymes	>gi|512917383|ref|XP_00492	ecdysone oxidase-like protein 2	_	_	36	36	36
ecdysone metabolism enzymes	>BGIBMGA010515-PA pep	ecdysone oxidase-like protein 3	_	_	34	34	13
ecdysone metabolism enzymes	>BGIBMGA005710-PA pep	ecdysone oxidase-like protein 4	_	_	29	28	28
ecdysone metabolism enzymes	>gi|512938073|ref|XP_00493	ecdysone oxidase-like protein 5	_	_	32	11	10
ecdysone metabolism enzymes	>BGIBMGA013006-PA pep	ecdysone oxidase-like protein 7	_	Yes	27	27	27
ecdysone metabolism enzymes	>BGIBMGA005711-PA pep	ecdysone oxidase-like protein 6	_	_	17	15	15
ecdysone metabolism enzymes	>BGIBMGA009925-PA pep	ecdysone oxidase-like protein 8	_	_	7	7	7
ecdysone metabolism enzymes	>BGIBMGA013193-PA pep	3-dehydroecdysone 3 alpha-reductase	_	_	4	4	4
ecdysone metabolism enzymes	>BGIBMGA009799-PA pep	3-dehydroecdysone 3 beta-reductase-like protein 1	_	_	21	21	19
ecdysone metabolism enzymes	>gi|512901370|ref|XP_00492	3-dehydroecdysone 3 beta-reductase-like protein 2	_	_	9	9	9

Sequence coverage [%]	Mol. weight [kDa]	PEP	Normalized iBAQ V-5 ASG1	Normalized iBAQ V-5 ASG2	Normalized iBAQ V-5 ASG3	Normalized iBAQ V-5 A-MSG1	Normalized iBAQ V-5 A-MSG2	Normalized iBAQ V-5 A-MSG3	Normalized iBAQ V-5 M-MSG1	Normalized iBAQ V-5 M-MSG2	Normalized iBAQ V-5 M-MSG3	Normalized iBAQ V-5 P-MSG1	Normalized iBAQ V-5 P-MSG2	Normalized iBAQ V-5 P-MSG3	Normalized iBAQ V-5 PSG1	Normalized iBAQ V-5 PSG2
53.5	63.296	0	0.03989	0.04793	0.04229	0.15424	0.0975	0.11679	0.23829	0.24306	0.19372	0.0188	0.01722	0.01489	0.00129	0.00127
42.4	61.415	4.99E-142	0.02617	0.0179	0.02378	0.00118	0.00066	0.00103	0.00027	0.00243	0.00036	0.0002	0.00011	0.00011	0	0
40.2	61.701	0	0.49486	0.57272	0.58266	0.08478	0.07225	0.09019	0.00643	0.00897	0.0083	0.00012	0	0	0	0
55.3	64.261	0	2.53434	2.72132	2.49368	0.47938	0.42997	0.7226	0.00232	0.00178	0.002	0	0	0	0	0
32.9	63.58	0	0.01095	0.01039	0.0116	0	0	0.00046	0	0	0	0	0	0	0	0
48.7	52.711	3.42E-111	0.00435	0.00316	0.0054	0.00317	0.00141	0.00433	0.01533	0.00862	0.01367	9.3E-05	0.00011	9E-05	0	0
27.5	52.886	3.42E-142	0.00085	0.00109	0.00119	0	0	0	0	0	0	0	0	0	0	0
58.6	61.127	3.65E-276	0	0	0	0	0	0.00023	0.03589	0.03425	0.02598	0.02828	0.02775	0.03243	0.00795	0.00803
23.6	59.35	3.58E-27	0	0	0	0	0	0	0.0015	0.00054	0.00133	0.00211	0.0016	0.00184	0.00206	0.00216
14.2	84.821	7.83E-62	0	0	0.0001	0.00014	0	0.00011	0.00018	0	0	4.6E-05	0	0	0	0
8.8	64.224	1.91E-12	0	0	0	0.0003	0	0	0	0	0	0.00011	0.00013	9.9E-05	0	0
10.6	52.496	8.05E-06	0	0	0	0	0	0	0	0	0	0	0	0	0	0
35.9	57.55	0	1.53611	1.61172	1.60661	0.46492	0.5026	0.62803	0.0007	0.00047	0.00077	0	0	0	0	0
52.3	22.373	7.66E-45	0	0	0.00043	0	0	0	0	0	0	0.00285	0.00115	0.00175	0.01106	0.01433
73.9	61.805	0	1.82009	1.75792	1.86632	1.052	0.97364	1.21553	0.00907	0.00726	0.00975	0	0	0	0	0
63.2	66.855	0	1.36287	1.35309	1.44854	0.56962	0.47219	0.46693	0	0	0	0	0	0	0	0
58	57.577	0	0	0	0	0	0	0	0	0	0	0	0	0	0	0
52	65.475	2.84E-292	0	0	0	0	0	0	0.00611	0.0015	0.00426	0.00515	0.00366	0.00352	0	0
60	57.655	0	0	0	0	0	0	0	0	0.00012	0.00011	0.00033	0.00016	0.00017	0	0
27.9	139.1	1.35E-177	0	0	0	0	0	0	0	0	0	0	0	0	0	0
37.9	62.784	2.04E-106	0	0	0	0	0	0	0	0	0	0	0	0	0	0
11.6	69.366	2.36E-11	0	0	0	0	0	0	0	0	0	0	0	0	0	0
19.3	26.916	4.39E-19	0	0	0	0	0	0	0	0	0	0	0	0	0	0
38.4	41.843	4.28E-105	0	0	0	0	0	0	0	0	0	0	0	0	0	0
30.6	35.835	2.52E-17	0	0	0	0	0	0	0	0	0	0	0	0	0.00031	0.00022

**Normalized iBAQ V-5 PSG3**	**Normalized iBAQ W-1 ASG1**	**Normalized iBAQ W-1 ASG2**	**Normalized iBAQ W-1 ASG3**	**Normalized iBAQ W-1 A-MSG1**	**Normalized iBAQ W-1 A-MSG2**	**Normalized iBAQ W-1 A-MSG3**	**Normalized iBAQ W-1 M-MSG1**	**Normalized iBAQ W-1 M-MSG2**	**Normalized iBAQ W-1 M-MSG3**	**Normalized iBAQ W-1 P-MSG1**	**Normalized iBAQ W-1 P-MSG2**	**Normalized iBAQ W-1 P-MSG3**	**Normalized iBAQ W-1 PSG1**	**Normalized iBAQ W-1 PSG2**	**Normalized iBAQ W-1 PSG3**
0.00084	0.39665	0.29785	0.33903	0.83836	0.91176	1.09019	0.19231	0.24156	0.19603	0.00028	0.0002	0.00021	6.3E-05	0	8.2E-05
0	0.00012	0.00013	0.00042	0.00011	0.00034	0.00036	0.00028	0.00028	0.00025	0	0	0	0	0	0
0	0.00675	0.00599	0.00442	0.00176	0.00091	0.00114	0	0	0.00026	0	0	0	0	0	0
0	0.00598	0.00141	0.00308	0	0	7E-05	0	0	0	0	0	0	0.0003	0.00059	0.00036
0	0.01707	0.01271	0.0146	0.00433	0.00579	0.0037	0	0	0	0	0	0	0	0	0
0	0.01009	0.01169	0.01121	0.00254	0.00493	0.00659	0.00061	0.00147	0.00032	0	0	0	0	0	0
0	0.00344	0.00454	0.0029	0	0.00053	0.0018	0	0	0	0	0	0	0	0	0
0.00992	0.01971	0.01808	0.02007	0.0119	0.01477	0.01644	0.0062	0.00883	0.00811	0	0	0	0	0	0
0.00171	9.2E-05	0	0	0	0	0.00187	0	0	0	0	0	0	0.00141	0.00065	0.00159
0	0	0	0	0.00138	0.00154	0.00256	0	0	0	0	0	0	0	0	0
0	0	0	0	0	0	0	0	0	0	0	0	0	0	0	0
0	0	0	0	0	0.00012	0.00014	0	0	0	0	0	0	0	0	0
0	0	0	0	0	0	0	0	0	0	0	0	0	0	0.00035	0
0.0153	0	0.00136	0	0.00038	0	0	0	0	0	0	0	0	0.0169	0.0176	0.01286
0	0	0	0	0	0	0	0	0	0	0	0	0	0.00019	0.00012	0
0	0.00222	0.00308	0.00238	0.00017	0.0002	0.00015	0	0	0	0	0	0	0.00013	0.00031	0.00012
0	0.11961	0.13169	0.12402	0.0571	0.05118	0.06227	0	0	0	0	0	0	0	0	0
0	0.00137	0.00116	0.00113	0.01795	0.01374	0.0262	0.00771	0.00444	0.00401	0	0	0	0	0	0
0	0.00884	0.00818	0.00793	0.00473	0.00433	0.0048	0.00093	0	0.00209	0	0	0	0	0	0
0	0.00016	0.00061	4.5E-05	0.00019	0.00021	3.4E-05	0.00316	0.00141	0.00127	0.00027	0.00039	0.00024	0	0	0
0	0	0	0	6.4E-05	0	0	0.00697	0.00116	0.00421	0	0	0	0	0	0
0	7.8E-05	0.00021	0.00032	8.5E-05	0	0	0	0	0	0	0	0	0	0	0
0	0.00321	0.0009	0.00205	0.00163	0.0029	0.00192	0	0	0	0	0	0	0	0	0
0	0.04946	0.04059	0.02765	0.04815	0.05443	0.11589	0.00101	0	0	0	0	0	0.00028	0.00062	0.00101
0.00073	0.0007	0.00078	0.00099	0.001	0.00087	0.00161	0	0	0	0	0	0	0.00086	0.00101	0.00098

Supplementary Table S3. Primer sequences, sizes of PCR products and melting temperature for semi-quantitative RT-PCR of hormone metabolism enzymes in the silk gland lumen.

Gene	(5'-3')	Primer	Size(bp)	Melting temperature(℃)
BGIBMGA013930	Forward	AAGCCATTCCTCATCTCAC	319	54
	Reverse	AATAAGGCACCGACAAACT		
BGIBMGA005322	Forward	ATAATGTTCAGTGGGAGTG	550	54
	Reverse	TATGCGTGAATGCTATGTC		
BGIBMGA013812	Forward	GATGAAGGACCAAGTAGCC	301	58
	Reverse	CCAATCTTTAGCCATGAGAC		
BGIBMGA012031	Forward	AGAGTCCACAACCACCAG	361	54
	Reverse	ACTTGATCCTTCAGTCCC		
BGIBMGA007546	Forward	CCTTTACCACCGCCAACA	503	56
	Reverse	CCGCTACCTGCGATAACC		
BGIBMGA000776	Forward	GAAACCACCCACAGGAGC	441	56
	Reverse	CTTTGAACCCAGCGTAGC		
BGIBMGA009211	Forward	CTTAGATGGCTGGCTTCG	647	56
	Reverse	GAGTATCAGGCTTAGTGGC		
BGIBMGA010516	Forward	CATCCGAGGGAACAGACA	791	54
	Reverse	TACCAACGGGAAACACGA		
BGIBMGA010515	Forward	GTTACGCACTACCGACAT	522	52
	Reverse	TACCAACGGGAAATACG		
BGIBMGA013193	Forward	TGAAGAAGACCGTGGAAC	332	54
	Reverse	GGCGTTTACTCTTATCCC		
BGIBMGA009799	Forward	CGGGAAGACCGTCACTCA	200	58
	Reverse	CGTAGGGCTGCCAGAACA		
BGIBMGA012152	Forward	CCATTGACATTGGCTACCG	303	56
	Reverse	CGTCCGAGAACTGGATTTT		
BGIBMGA005710	Forward	TAGCATCGGCTGTGGTCT	530	56
	Reverse	TGCGGTCAGGAAACTCGTA		
BGIBMGA012872	Forward	TGAGCGAGAAACACCACT	644	52
	Reverse	GTCCGCCAAACTTGAATA		
BGIBMGA000158	Forward	TTGAAGCAGGAACATACCC	395	54
	Reverse	CAATAAGTTGTGCGTGGC		
